# Expression of sterile-α and armadillo motif containing protein (SARM) in rheumatoid arthritis monocytes correlates with TLR2-induced IL-1β and disease activity

**DOI:** 10.1093/rheumatology/keab162

**Published:** 2021-02-19

**Authors:** Ryan S Thwaites, Sarah Unterberger, Giselle Chamberlain, Henry Gray, Kelsey Jordan, Kevin A Davies, Neil A Harrison, Sandra Sacre

**Affiliations:** 1 Brighton and Sussex Medical School, University of Sussex; 2 Rheumatology Department, The Royal Sussex County Hospital, Brighton and Sussex University Hospitals NHS Trust, Brighton, UK

**Keywords:** rheumatoid arthritis, toll-like receptor, interleukin-1, sterile-α and armadillo motif containing protein (SARM), DAS28

## Abstract

**Objective:**

Cartilage and bone damage in RA are associated with elevated IL-1β. The effects of IL-1β can be reduced by biological therapies that target IL-1β or TNF-α. However, the mechanisms responsible for increased IL-1β and the effect of anti-TNF-α have not been fully elucidated. Recently, sterile-α and armadillo motif containing protein (SARM) was identified as a negative regulator of toll-like receptor (TLR) induced IL-1β secretion through an interaction with the inflammasome. This study set out to investigate SARM during TLR-induced IL-1β secretion in RA peripheral blood monocytes and in patients commencing anti-TNF-α treatment.

**Methods:**

Monocytes were isolated from RA patients and healthy controls; disease activity was measured by DAS28. IL-1β secretion was measured by ELISA following TLR1/2, TLR4 and TLR7/8 stimulation. The mRNA expression of *SARM1*, IL-1β and the components of the NOD-like receptor family pyrin domain containing 3 (NLRP3) inflammasome were measured by quantitative PCR. SARM protein expression was measured by western blotting.

**Results:**

TLR1/2 activation induced elevated IL-1β in RA monocytes compared with healthy controls (*P* = 0.0009), which negatively correlated with *SARM1* expression (*P* = 0.0086). Lower SARM expression also correlated with higher disease activity (*P* = 0.0246). Additionally, patients responding to anti-TNF-α treatment demonstrated a rapid upregulation of SARM, which was not observed in non-responders.

**Conclusion:**

Together, these data highlight a potential contribution from SARM to RA pathophysiology where decreased SARM may lead to elevated IL-1β associated with RA pathogenesis. Furthermore, the data additionally present a potential mechanism by which TNF-α blockade can modify IL-1β secretion.

**Rheumatology key messages**

- *SARM1* negatively correlates with TLR2-induced IL-1β and RA disease activity.
- RA patients with active disease express lower levels of SARM.
- Patients that responded to anti-TNF-α therapy exhibit an increase in SARM expression.

## Introduction

RA is a chronic autoimmune disease associated with persistent inflammation of the synovial joints. Elevated production of pro-inflammatory cytokines leads to articular damage, together with cardiovascular, metabolic and neurological comorbidities [1]. IL-1β, TNF-α and IL-6 are key cytokines involved in sustaining RA pathogenesis. Targeting these cytokines with biological therapies can reduce inflammation and suppress joint damage [2]. The biologics that target TNF-α are the most widely used as treatments for RA and they can also result in a reduction in IL-1β [3–5]. However, direct inhibition of IL-1β has proven to be less effective than targeting TNF-α, producing only moderate benefit, so tends to be rarely used for clinical treatment of RA. Although, IL-1β does not appear to have a pivotal role in controlling inflammation in RA, it contributes to synovial joint damage and may have a role in comorbidities such as type 2 diabetes and cardiovascular disease [6]. Within the joint, IL-1β activates osteoclasts and chondrocytes leading to cartilage erosion and bone resorption [7].

Monocytes recruited from the peripheral blood that differentiate into synovial tissue macrophages are considered to be the main source of IL-1β [8]. The secretion of IL-1β involves several processes; transcriptional upregulation of pro-IL-1β is first induced in response to cell stimulation, followed by proteolytic cleavage of pro-IL-1β by caspase-1, a component of the nucleotide-binding oligomerization domain (NOD)-like receptor family pyrin domain containing 3 (NLRP3) inflammasome, to produce biologically active IL-1β [9]. Caspase-1, once activated, additionally cleaves gasdermin D, freeing the N-terminus to oligomerize forming a pore in the cell membrane, permitting the release of IL-1β and inducing pyroptosis [10, 11]. Further regulation comes from sterile-α and armadillo motif containing protein (SARM) that negatively regulates the assembly of the NLRP3 inflammasome, suppressing the maturation of IL-1β [12]. Murine cells and human macrophages require two separate signals to produce mature IL-1β, one to induce transcription of pro-IL-1β and a second signal to activate the inflammasome. However, human monocytes can secrete IL-1β through toll-like receptor (TLR) activation alone in a pyroptosis-independent manner [13].

A potential pathway for the induction of IL-1β in RA is through activation of TLRs. This family of pattern recognition receptors has been suggested to contribute to the chronic inflammation characteristic of both human disease and experimental animal models of RA [14]. Indeed, the RA joint contains many endogenous TLR ligands such as tenascin-C and ACPA immune complexes containing citrullinated fibrinogen, which can activate TLR4 [15, 16]. Furthermore, ACPAs purified from RA patient serum can additionally induce NLRP3-dependent IL-1β release from human granulocyte-macrophage colony-stimulating factor-derived macrophages [17].

Although many studies have demonstrated a contribution of IL-1β to RA pathogenesis, the underlying processes leading to increased IL-1β production in RA remain less well defined. The aim of this study was to investigate the relationship of SARM to IL-1β secretion in RA pathogenesis. SARM expression was examined in peripheral blood monocytes prior to stimulation with TLR ligands. RA patients with active disease expressed lower levels of SARM compared with healthy volunteers. *SARM1* expression inversely correlated with RA disease activity and IL-1β release upon TLR1/2 activation. Furthermore, RA patients who responded to anti-TNF-α therapy exhibited a transient increase in SARM that was not observed in non-responders, providing a possible mechanism by which TNF-α blockade additionally reduces IL-1β levels in RA patients. Together, these data support a role for SARM in RA pathophysiology where decreased SARM may contribute to the elevated level of IL-1β associated with RA pathogenesis.

## Methods

### Patient samples

The study was approved by the Brighton East Research Ethics Committee (10/H1107/8), National Research Ethics Service (NRES) committee South East Coast–Brighton and Sussex (11/LO/1320) and the NRES Committee North West–Lancaster (14/NW/1114). Patients were diagnosed by a consultant rheumatologist guided by the EULAR/ACR 2010 criteria for RA and recruited during a routine rheumatology clinic assessment. Healthy controls (HCs) were recruited through the Brighton and Sussex University Hospitals Trust. The study complies with the Declaration of Helsinki. All participants provided informed written consent. Characteristics of the donors included throughout this study are summarized in Table 1. Samples were collected over a 10-year period, consequently the number of RA patients receiving biological therapies within this study are lower than would be observed in current clinical practice.

**Table 1:** Demographics, clinical data and treatment modalities of study participants

Characteristics	Value
**Healthy controls, *n***	27
Age, years, mean (s.d.)	44.6 (11.53)
Sex, male:female	3:23 (1 unknown)
**RA, *n***	99
Age, years, mean (s.d.)	57.48 (15.06)
Sex, male:female	24:75
DAS28, mean (s.d.)	4.51 (1.83)
Disease duration, years, mean (s.d.)	12.36 (12.76)
**Medication of RA patients**	
csDMARDs	72 (72.7)
Methotrexate	41 (41.4)
Other csDMARDs (SSZ, HCQ, AZA, LEF)	18 (18.2)
Methotrexate and other csDMARDs	19 (19.2)
bDMARDs	14 (14.1)
TNF-α inhibitor	11 (11.1)
Other bDMARDs (IL-6 inhibitor, anti-CD20)	2 (2.0)
TNF-α inhibitor and anti-CD20	1 (1.0)
NSAIDs	10 (10.1)
Glucocorticoids	26 (26.3)
Antidepressants	1 (1.0)
Analgesics	1 (1.0)
No or unknown treatment	16 (16.2)

Data are *n* (%) except where indicated. bDMARDs: biological disease-modifying antirheumatic drugs; csDMARDs: conventional synthetic disease-modifying antirheumatic drug; DAS28: disease activity score 28.

### Cell culture

Whole venous blood was collected into tubes containing 1.8 mg/ml (K2) EDTA (Becton Dickinson, Oxford, UK) and stored at room temperature for up to 2 hours prior to cell separation. Leucocyte cones from blood donors were purchased from NHS Blood and Transplant (Tooting, UK) to determine *SARM* expression upon TLR activation . Peripheral blood mononuclear cells (PBMCs) were isolated using Ficoll-Paque gradients (Cedarlane, Burlington, Canada) as previously described [18]. Monocytes from PBMCs derived from whole venous blood were isolated using CD14^+^ selection beads (Miltenyi Biotec, Cologne, Germany) as per the manufacturer’s instructions. Peripheral blood monocytes from PBMCs derived from leucocyte cones were isolated by iso-osmotic Percoll gradient centrifugation, as previously described [19], before being cultured in RPMI1640 media supplemented with 5% (v/v) foetal calf serum and 1% (v/v) penicillin/streptomycin solution (PAA, Pasching, Austria) with or without 100 ng/ml PAM_3_CSK_4_ (Axxora, Nottingham, UK), 10 ng/ml lipopolysaccharide (LPS) (Axxora, Nottingham, UK) or 2 μg/ml resiquimod (R-848) (Enzo Life Sciences, Lausen, Switzerland) at 37°C, 5% CO_2_. All TLR ligands were used at predetermined concentrations that induce maximal cytokine induction.

### Quantitative polymerase chain reaction

RNA was extracted from CD14^+^ monocytes using a miRNeasy Mini Kit (QIAGEN, Stockach, Germany) according to manufacturer’s instructions and reverse transcribed using a High Capacity cDNA Reverse Transcription kit (Applied Biosystems, Paisley, UK) according to manufacturer’s instructions. The cDNA was analysed using Taqman quantitative PCR assays (Life Technologies,Carlsbad, USA) using Taqman PCR master mix 2x (Thermo Fisher Scientific, Waltham, MA, USA). The level of *SARM1* (assay: Hs00248344_m1), *PYCARD* (Hs00203118_m1), *CASP1* (Hs00354836_m1), *NLRP3* (Hs00918082_m1) and *IL1B* (Hs00174097_m1) were determined relative to the geometric mean of the housekeeping genes *GAPDH* (Hs02758991_g1) and *HPRT1* (Hs02800695_m1). Reactions were performed using a Stratagene MX3000P thermocycler or an Agilent AriaMX thermocycler (Agilent Technologies, Cheshire, UK) as per manufacturer’s instructions. Agilent Aria 1.6 software (Agilent Technologies) was used for data collection and analysis. The comparative threshold cycle method (2^-ΔCt^) was used for quantification of gene expression.

### Enzyme linked immunosorbent assays

The concentration of IL-1β in cell culture supernatants was determined using an ELISA DuoSet (R&D Systems, Abingdon, UK) following the manufacturer's instructions. Recombinant IL-1β standard was purchased from PeproTech (London, UK). Detection was performed using streptavidin-Horseradish Peroxidase (R&D systems, Minneapolis, USA) and a chromogenic 3,3′,5,5′-Tetramethylbenzidine (TMB) substrate (KPL, Gaithersburg, USA). Absorbance was read and analysed at 450 nm on a spectrophotometric plate reader (BioTek Synergy HT, Winooski, USA) using the Gen5 version 1.08.4 software.

### Western blotting

Monocytes were lysed in NP-40 buffer and protein concentrations were determined using bicinchoninic acid (BCA) protein assay kit (Life Technologies, Paisley, UK) according to the manufacturer’s instructions. Proteins were boiled in Laemmli buffer at 95°C for 5 minutes before resolved by SDS–PAGE using 10% Tris-glycine gels and transferred onto methanol-activated Polyvinylidene difluoride membranes (Life Technologies) After transfer, membranes were blocked with 3–5% BSA in PBS with 0.1% Tween-20 (PBS-T; Fisher Scientific, Loughborough, UK) for 1 hour at room temperature, and incubated overnight at 4°C with the following primary antibodies diluted in 3–5% BSA in PBS-T; anti-SARM at 1:1000 (ProSci, Poway, USA), and anti-GAPDH at 1:3000 (Cell Signalling, London, UK). A secondary anti-rabbit HRP-conjugated antibody (Sigma-Aldrich, Gillingham, UK) was used for detection. For protein visualization, blots were incubated with Amersham ECL Prime Western Blotting Detection Reagent (GE Healthcare, Little Chalfont, UK). Membranes were then removed from the ECL solution and exposed to photographic film (GE Healthcare, Little Chalfont, UK) and developed using a Konica Minolta SRX-101A film developer and Champion Photochemistry film developer and fixer solutions (Jet X-ray, London, UK).

### Statistics

Mean, s.d., s.e.m. and statistical significance were calculated using GraphPad version 8 (GraphPad Software Inc., California, USA). For statistical analysis, parametric data were compared using a two-tailed unpaired *t* test with Welch's correction, or a two-tailed paired *t* test. Non-parametric data were compared with a two-tailed Mann–Whitney *U* test. One-way ANOVA using Dunnett's multiple comparisons test was applied for the comparison of multiple datasets relative to a control for parametric distributed variables. Correlation analysis was performed using a Pearson two-tailed test for parametric data and the Spearman’s test for non-parametric data. SEM was used for pooled experimental data. Multivariable linear regression analysis was performed in SPSS version 26 (SPSS Inc., Chicago, IL, USA). Significance is shown as ****P < 0.0001, ***P *<* 0.001, **P *<* 0.01 and *P *<* 0.05.

## Results

### IL-1β production was elevated from RA monocytes upon TLR1/2 activation

**Fig. 1:**
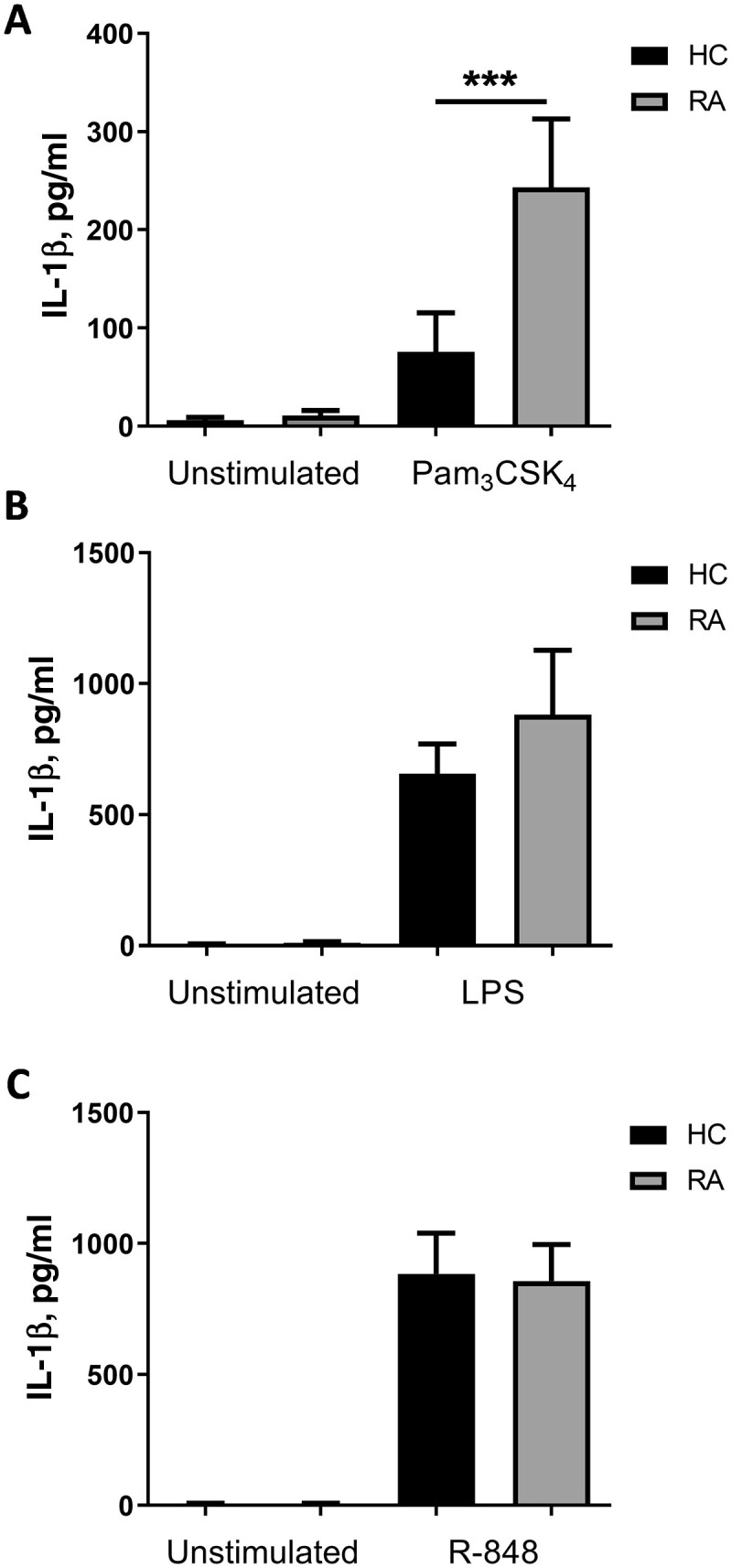
TLR1/2 activation induces higher IL-1β secretion from RA patient monocytes than healthy controls CD14^+^ monocytes were isolated from peripheral blood donated by HC and RA patients. IL-1β secretion was measured following 24 hours of culture with media alone (unstimulated) and (**A**) 100 ng/ml Pam_3_CSK_4_ (HC *n* = 18; RA *n* = 28), (**B**) 10 ng/ml LPS (HC *n* = 21; RA *n* = 29) or (**C**) 2 μg/ml R-848 (HC *n* = 22; RA *n* = 28). Data is shown as the mean ± SEM. Significance was tested using two-tailed Mann–Whitney *U* test (***P = 0.0009). HC: healthy control; LPS: lipopolysaccharide; TLR: Toll-like receptor.

To assess the capability of TLRs to induce IL-1β from RA monocytes, peripheral blood CD14^+^ monocytes from RA patients and HCs were stimulated with Pam_3_CSK_4_ to activate the TLR1/2 heterodimer, LPS to stimulate TLR4 and R-848, a ligand that can activate both TLR7 and TLR8 (Fig. 1). A significant elevation of IL-1β secretion was observed following TLR1/2 stimulation with Pam_3_CSK_4_ from RA monocytes relative to HCs (*P* = 0.0009; Fig. 1A) but surprisingly this was not observed from cells activated with LPS or R-848 (Fig. 1B and C). The elevated IL-1β upon TLR1/2 activation was not associated with treatment of either methotrexate or glucocorticoids (Supplementary Fig. S1A, available at *Rheumatology* online).

### *SARM1* expression was elevated in RA monocytes and inversely correlated with the level of TLR1/2-induced IL-1β release

**Fig. 2:**
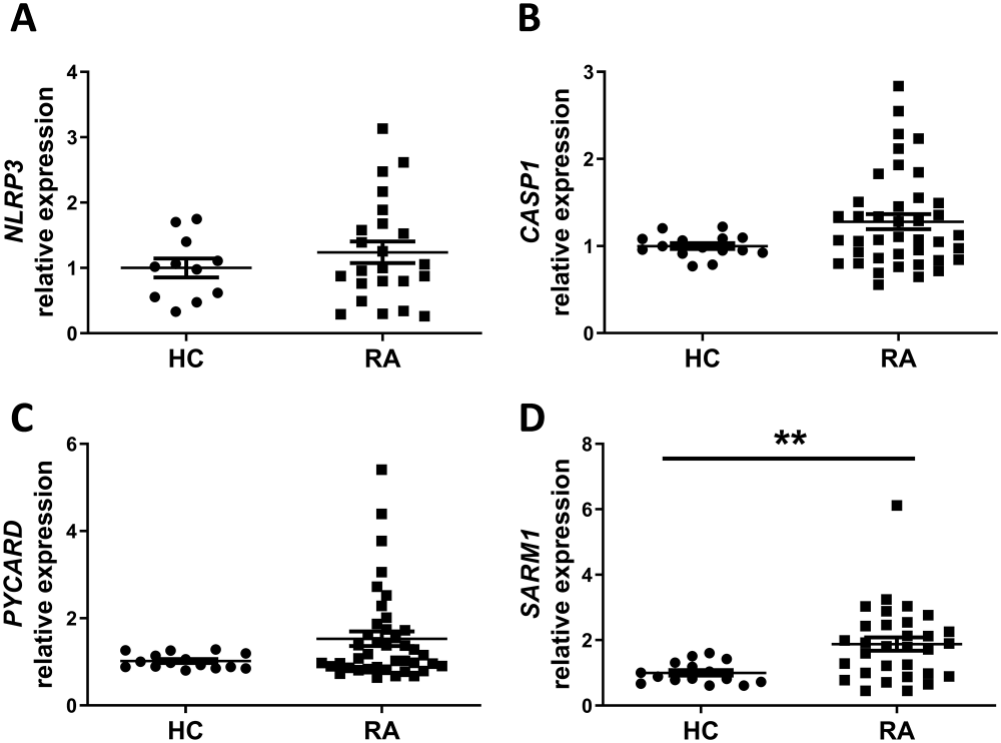
SARM mRNA expression is elevated in RA monocytes Monocytes were isolated from whole blood collected from healthy controls (HC) and RA patients. RNA was extracted and the expression of (**A**) *NLRP3* (HC *n* = 11; RA *n* = 23), (**B**) *CASP1* (HC *n* = 15; RA *n* = 40), (**C**) *PYCARD* (HC *n* = 15; RA *n* = 40) and (**D**) *SARM1* (HC *n* = 15; RA *n* = 31) were measured by quantitative PCR. Data is shown as the mean ± SEM. Significance was tested using two-tailed Mann–Whitney *U* tests (**P < 0.0054). SARM: sterile-α and armadillo motif containing protein.

To explore this enhancement of IL-1β secretion from RA monocytes, the gene expression of inflammasome components NLRP3 (gene name *NLRP3*)*,* pro-caspase-1 (gene name *CASP1*), apoptosis-associated speck-like protein containing a caspase recruitment domain (ASC; gene name *PYCARD*) and the NLRP3 inhibitor SARM (gene name *SARM1*) were measured. Equivalent expression of *NLRP3*, *CASP1* and *PYCARD* were observed between HC and RA monocytes (Fig. 2A–C, respectively). By contrast, increased expression of *SARM1* was evident in monocytes from RA patients relative to HC (*P* = 0.0054; Fig. 2D). The level of *SARM1* expression was not associated with treatment of either methotrexate or glucocorticoids (Supplementary Fig. S1B, available at *Rheumatology* online). However, a wide range of expression was observed within the RA samples for all of these genes, with some donors exhibiting similar levels of expression to HCs whilst others demonstrated a substantially increased expression.

**Fig. 3:**
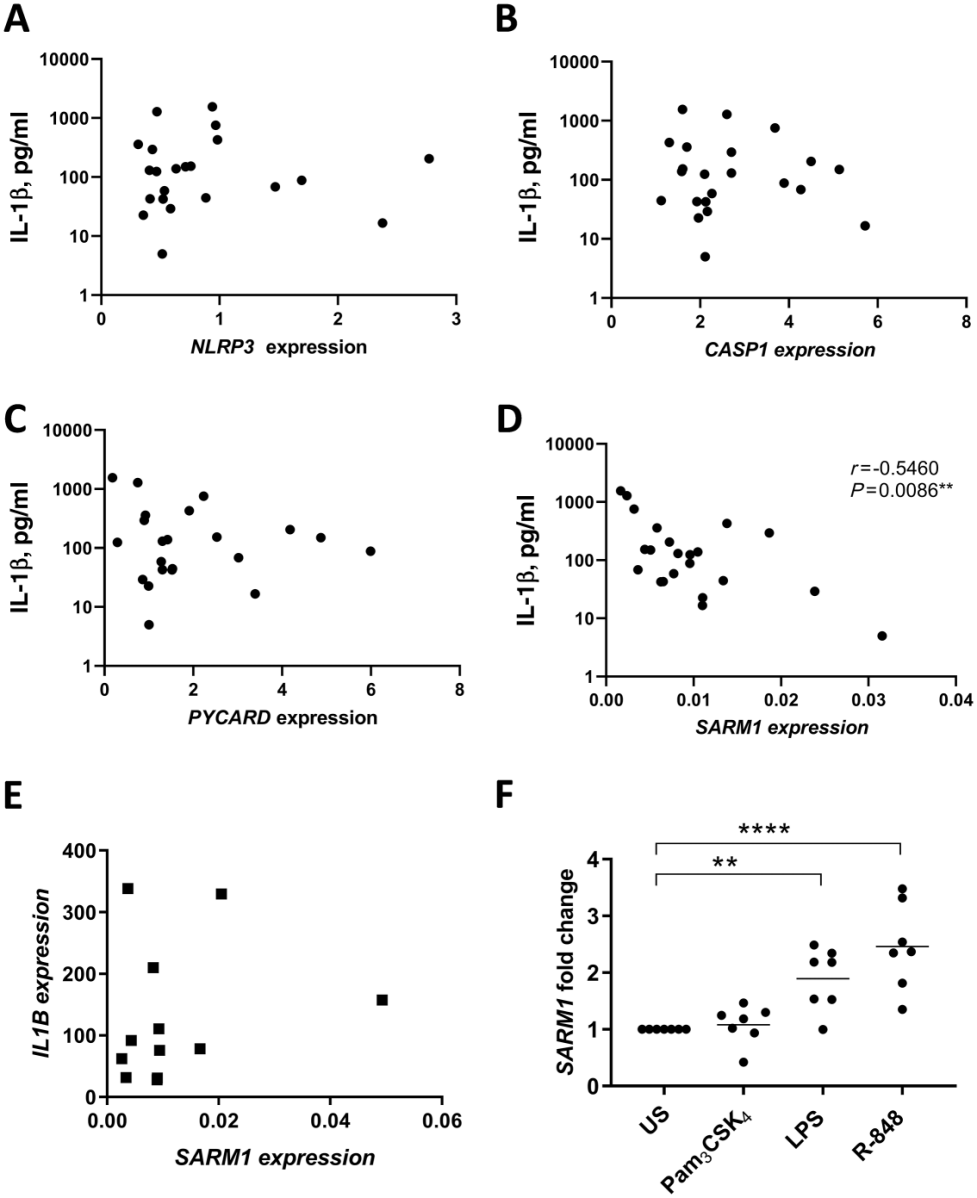
SARM expression negatively correlates with IL-1β secretion following TLR1/2 stimulation in RA monocytes TLR1/2-induced IL-1β secretion was measured following 24 hours of stimulation with 100 ng/ml Pam_3_CSK_4_ and correlated with the basal expression of (**A**) *NLRP3* (*n* = 22), (**B**) *CASP1* (*n* = 22), (**C**) *PYCARD* (*n* = 22) and (**D**) *SARM1* (*n* = 22) in monocytes from RA patients. The significance was analysed using a two-tailed Spearman test. (**E**) Monocyte *IL1B* expression following a 3-hour stimulation with 100 ng/ml Pam_3_CSK_4_ was compared with basal *SARM1* expression (prior to stimulation) in matched patient samples (*n* = 12). (**F**) Monocytes from healthy volunteers (*n* = 7) were stimulated for 6 hours with 100 ng/ml Pam_3_CSK_4_, 10 ng/ml LPS or 2 µg/ml R-848. *SARM1* expression was expressed as the fold change from US cells and shown as the mean ± SEM. A one-way ANOVA using Dunnett’s multiple comparisons test was used to test significance compared with US cells (**P = 0.007, ****P < 0.0001). LPS: lipopolysaccharide; SARM: sterile-α and armadillo motif containing protein; TLR: Toll-like receptor; US: unstimulated.

These data were then compared with the TLR1/2-induced IL-1β secretion from RA patient monocytes to explore if the varying levels of gene expression correlated with TLR1/2-induced IL-1β. No association was observed between *NLRP3*, *CASP1* or *PYCARD* and IL-1β secretion (Fig. 3A–C). However, an inverse relationship was observed between the basal expression of *SARM1* in unstimulated cells and TLR1/2-induced IL-1β secretion (*P* = 0.0086, *R* = −0.546; Fig. 3D), which was not observed for TLR4 or TLR7/8-induced IL-1β secretion (Supplementary Fig. S2, available at *Rheumatology* online). Multivariable linear regression analysis confirmed the association of *SARM1* with the level of IL-1β secretion upon TLR1/2 activation (Supplementary Table S1, available at *Rheumatology* online).

### SARM was upregulated in response to activation of TLR4 and TLR7/8, but not TLR1/2

The study that identified SARM as a negative regulator of the NLRP3 inflammasome demonstrated that SARM controlled IL-1β maturation but not the production of pro-IL-1β (gene name *IL1B*) in murine bone marrow-derived macrophages [12]. To verify that SARM was regulating TLR1/2-induced IL-1β through this NLRP3-dependent action and not by an alternative mechanism, basal expression of *SARM1* was compared with the transcriptional expression of *IL1B* induced upon TLR1/2 activation. In agreement with the original study, *SARM1* did not correlate with *IL1B* expression (Fig. 3E). However, it remained unclear as to why TLR1/2 induced elevated IL-1β secretion, which correlated with *SARM1* in the RA monocytes, while TLR4 and 7/8 produced equivalent levels of IL-1β to HCs (Fig. 1). Negative regulators of inflammatory signalling cascades are often expressed in response to cell activation to restore the cell to a state of homeostasis [20]. To explore if *SARM1* was upregulated in response to TLR activation, monocytes from healthy donors were cultured with Pam_3_CSK_4_, LPS or R-848 to measure the fold change in *SARM1* following stimulation. Both LPS and R-848 significantly upregulated *SARM1* expression (*P* = 0.007 and *P* *<* 0.0001, respectively), while TLR1/2 stimulation did not significantly affect expression (Fig. 3F).

### Higher RA disease activity was associated with lower SARM expression

**Fig. 4:**
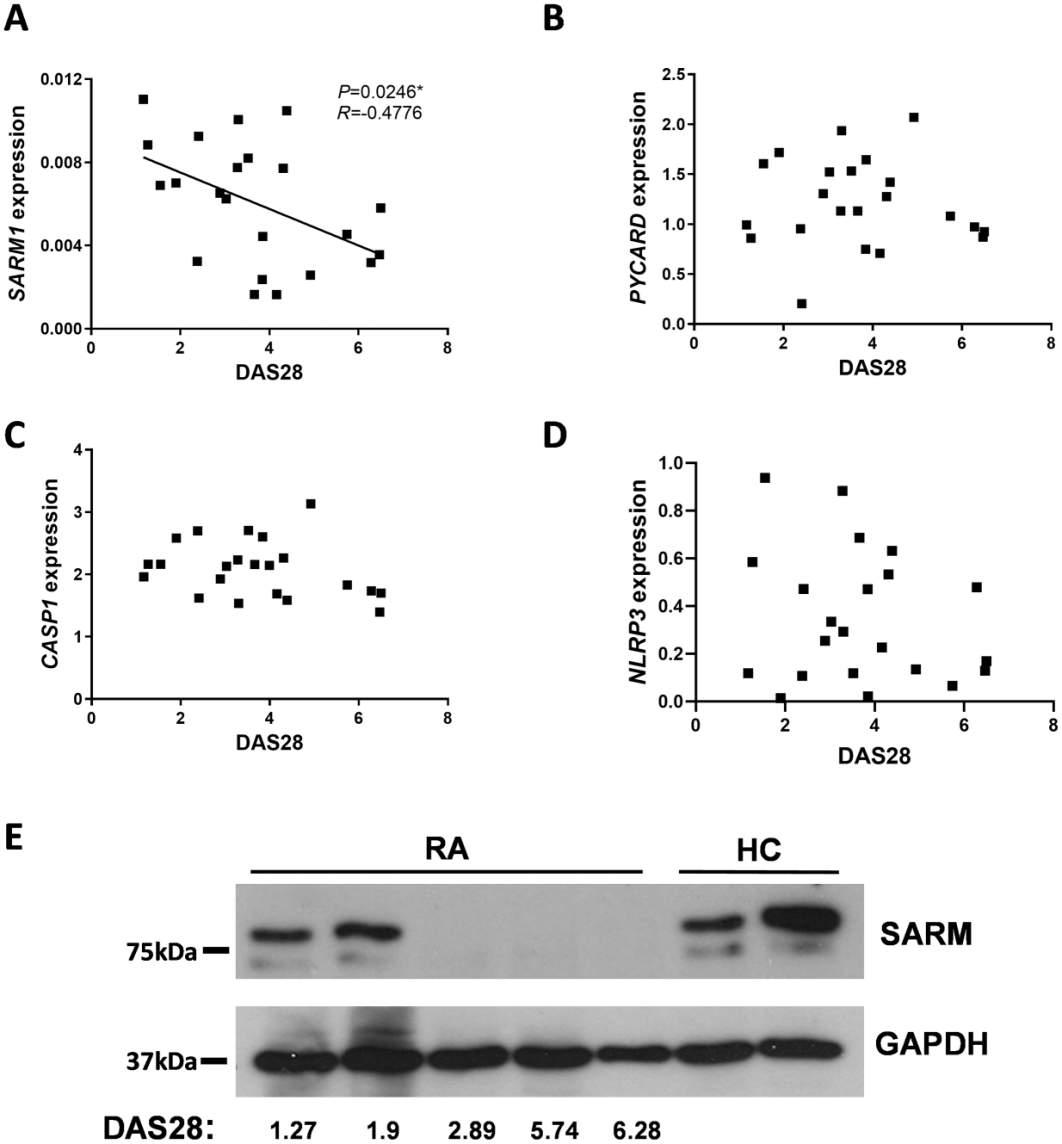
SARM expression by RA monocytes inversely correlates with disease activity Expression of (**A**) *SARM1* (*n* = 22), (**B**) *PYCARD* (*n* = 22), (**C**) *CASP1* (*n* = 22) and (**D**) *NLRP3* (*n* = 22) in monocytes isolated from RA patients correlated to the DAS28. Significance was analysed using a two-tailed Pearson’s test (*P = 0.0246). (**E**) SARM protein expression in monocytes from HC and RA patients with a range of DAS28 scores was analysed by western blot with GAPDH as a loading control. DAS28: disease activity score in 28 joints; HC: healthy controls; SARM: sterile-α and armadillo motif containing protein.

IL-1β has long been associated with RA pathogenesis leading to cartilage and bone erosion [21]. As lower *SARM1* expression was associated with elevated IL-1β release from RA monocytes (Fig. 3D), *SARM1* expression was next correlated with the disease activity score in 28 joints (DAS28), a clinical measure of RA disease activity [22]. Expression of *SARM1* in RA monocytes was inversely correlated with DAS28 (*P* = 0.0246, *R* = −0.4776; Fig. 4A). Multivariable linear regression analysis confirmed the association of *SARM1* with DAS28 (Supplementary Table S1, available at *Rheumatology* online). By comparison, no significant associations between DAS28 and expression of *PYCARD*, *CASP1* or *NLRP3* were observed (Fig. 4B–D). To confirm the relationship between *SARM1* and RA disease activity, SARM protein expression was analysed in monocyte lysates from HCs and RA patients over a range of DAS28 scores. DAS28 can be used to categorize RA disease activity scores into groups, with scores of <2.6 representing patients in remission, 2.6 to <3.2 as low disease activity, 3.2 to ≤5.1 as moderate disease activity and scores >5.1 as high disease activity [23]. SARM protein levels were similar between RA donors in disease remission and HCs; by comparison, SARM protein was not detected in monocytes from RA donors with active disease (Fig. 4E).

### Patients that respond to anti-TNF-α treatment demonstrated a rapid but transient increase in SARM expression

**Fig. 5:**
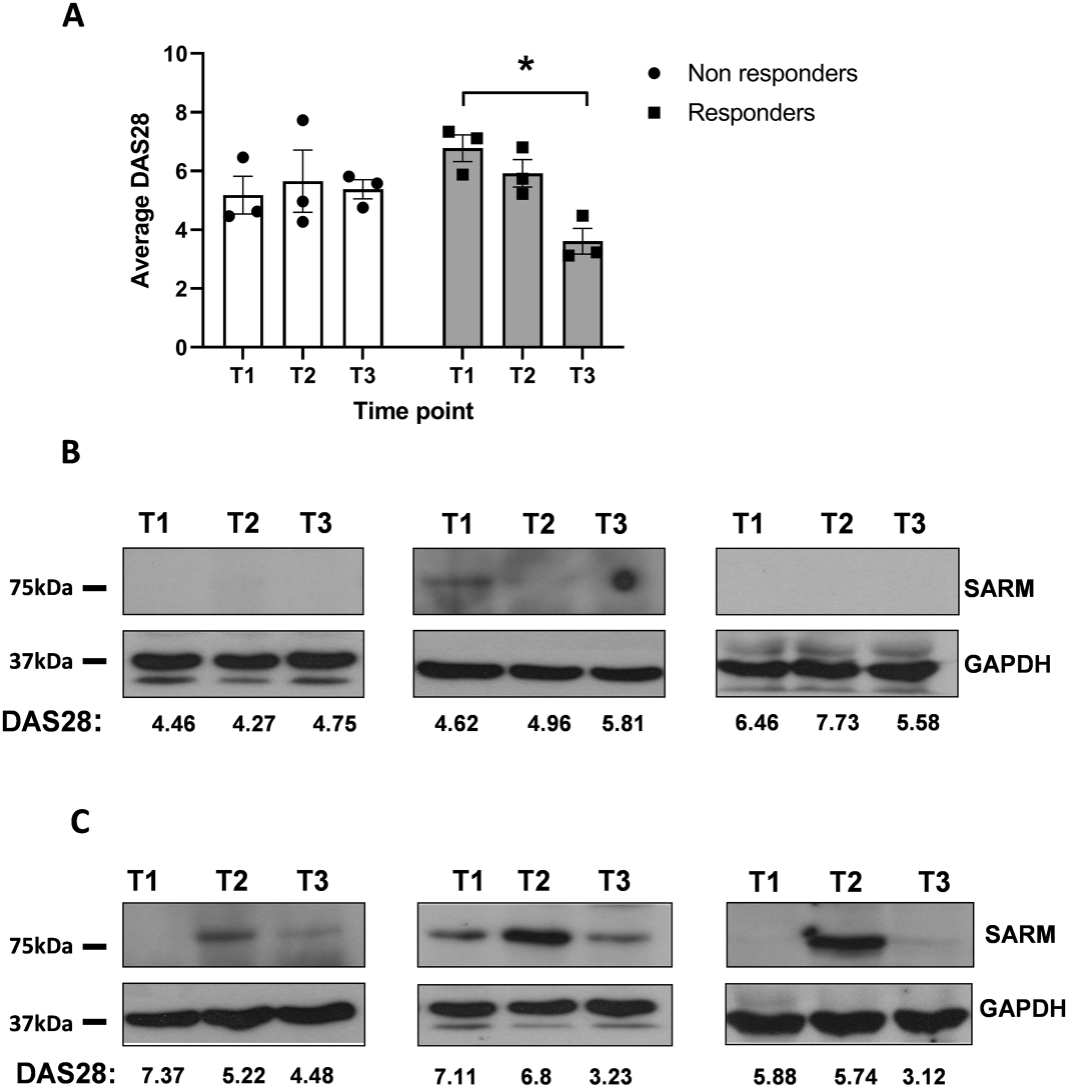
SARM protein expression is increased in patients that responded to anti-TNF-α therapy Patients starting anti-TNF-α treatment were monitored at three time points; pretherapy (T1), 24 hours post-therapy (T2) and 3 months post-therapy (T3). (**A**) At each time point the DAS28 was measured (*n* = 6). Data is shown as the mean ± SEM. A paired students *t* test was used to test significance (*P = 0.0125). The level of SARM protein expression was also measured by western blot at these time points in (**B**) patients that did not respond to the anti-TNF-α therapy (*n* = 3) and (**C**) in those that did respond with a reduction in DAS28 score (*n* = 3). GAPDH was used as a loading control. DAS28: disease activity score in 28 joints; SARM: sterile-α and armadillo motif containing protein.

As TNF-α blockade is reported to lower IL-1β in addition to neutralizing the effects of TNF-α, SARM protein expression during the initiation of anti-TNF-α biological therapy was investigated in RA peripheral blood monocytes [3, 4, 24]. Samples were collected at three time points, T1 (prior to anti-TNF-α treatment), T2 (24 hours following initiation of treatment) and T3 (3 months after initiation of anti-TNF-α treatment). Approximately one-third of RA patients do not respond to anti-TNF-α treatment [25]. Thus, the patient responses to treatment were recorded according to the EULAR response criteria [26]. Three patients displayed no response to treatment, whilst three showed an improvement by T3 (Fig. 5A). In the patients where no improvement of the DAS28 was evident, SARM expression was not increased between T1, T2 or T3 (Fig. 5B). However, for patients where a good or moderate response was detected, SARM was transiently increased at T2, which then declined at T3 (Fig. 5C).

## Discussion

Activation of the NLRP3 inflammasome and consequent downstream release of IL-1β could contribute to several aspects of RA pathogenesis. IL-1β has long been associated with bone and cartilage damage in RA joints, through activation of chondrocytes and osteoclasts [21]. However, more recently, IL-1β has also been suggested to have a role in RA comorbidities such as type 2 diabetes and cardiovascular disease [27]. In a recent, randomized controlled trial, anakinra (a human IL-1 receptor antagonist) was found to reduce the percentage of glycated haemoglobin in RA patients with type 2 diabetes [28]. Furthermore, improvements in cardiac function have also been reported in RA patients treated with anakinra [29].

Since the discovery of the NLRP3 inflammasome, a more detailed understanding of IL-1β production in RA has evolved. In the collagen induced arthritis model, treatment with MCC950, a selective inhibitor of NLRP3, reduces IL-1β, synovial inflammation and cartilage erosion [30]. In human studies, components of the NLRP3 inflammasome are reported to be increased in RA PBMCs and ACPAs present in the sera of some RA patients can stimulate NLRP3-dependent IL-1β release [17, 31]. Furthermore, incubation of RA synovial biopsies cultured with MCC950 is reported to reduce IL-1β [32]. However, the contribution of negative feedback mechanisms controlling activation of the NLRP3 inflammasome have yet to be examined in the context of RA pathophysiology.

Recently, SARM was identified as a negative regulator of NLRP3, inhibiting the recruitment of ASC and consequent inflammasome assembly [12]. Here, our study reveals an association between dysregulation of SARM expression in RA monocytes, elevated disease activity and increased IL-1β production. In keeping with its role as a regulator of IL-1β release, low *SARM1* expression in RA monocytes correlated with enhanced IL-1β secretion upon TLR1/2 stimulation with Pam_3_CSK_4_. However, increased IL-1β was not observed upon TLR stimulation with LPS or R-848 compared with HCs. Initially it was not clear why increased IL-1β was only evident upon TLR1/2 activation, as in murine bone marrow-derived macrophages SARM deficiency leads to an increase in IL-1β release upon activation with LPS or Pam_3_CSK_4_ [12]. However, upon further investigation of human monocytes, SARM was found to be upregulated at the message level in response to TLR stimulation with LPS and R-848 but not with Pam_3_CSK_4_.

This suggested a negative feedback role for SARM that was absent following TLR1/2 activation in human monocytes. Thus, RA monocytes from patients with active disease, which have a lower basal level of SARM compared with HC monocytes, could therefore release greater IL-1β upon TLR1/2 stimulation. Whereas upon stimulation with LPS or R-848, upregulation of SARM may compensate for this low basal expression, restoring IL-1β to a comparable level to HC monocytes. These data revealed the potential for TLR2 on monocytes to contribute to RA pathology through increased IL-1β production. This is in keeping with other studies suggesting a role for TLR2 in RA. TLR2 expression has been shown to be increased in both circulating monocytes and macrophages in the synovial joint [33]. We have previously demonstrated elevated TLR2 induced TNF-α and IL-6 from RA monocytes [34]. Also, activation of TLR2 in RA synovial fibroblasts leads to increased cell migration, invasion and glycolytic activity [35, 36]. Furthermore, addition of a TLR2 neutralizing antibody has been shown to reduce spontaneous cytokine production, including IL-1β from human RA synovial explants [37].

Investigation of the expression of the inflammasome components revealed that despite *NLRP3*, *CASP1* and *PYCARD* being reported to be increased in PBMCs from RA patients, this was not the case for the purified monocytes studied here [31]. Although both studies demonstrated a spread in the level of expression within the RA patient samples, in this study although a trend towards increased expression was observed, this did not reach significance. However, *SARM1* levels were significantly higher in the RA patients compared with HCs but, curiously, this did not equate to the presence of higher protein levels. *SARM1* expression did inversely correlate with RA disease activity but SARM protein was almost undetectable by western blot in most patients with active disease. However, monocytes from RA patients in remission (DAS28 < 2.6) exhibited a comparable level of SARM to HCs.

This discrepancy in the mRNA and protein expression may reflect post-transcriptional control, possibly by non-coding RNAs. Indeed, the finding that patients who were responsive to anti-TNF-α biologics had a transient increase in SARM may indicate a regulatory mechanism downstream of TNF-α. Inhibition of TNF-α in RA patients is reported to decrease the level of other inflammatory cytokines, including IL-1β, in addition to its effects on TNF-α [3–5]. Thus, the downstream regulation of SARM expression by TNF-α biologics in patients that respond to treatment may explain the additional suppressive effect on IL-1β. Biological therapies blocking TNF-α are far more effective in RA than those that inhibit the action of IL-1β, most likely due to an upstream role of TNF-α in regulating inflammation [6]. However, this does not preclude a functional role for IL-1β in RA pathogenesis. Indeed, a study in a human TNF transgenic arthritis model suggested that TNF-induced bone and cartilage damage may be mediated by IL-1 [38]. It will next be important to develop an understanding of how SARM protein expression is regulated in RA, as this may have relevance to RA comorbidities and other diseases where NLRP3 activation and excess IL-1β are associated with disease pathology, such as cardiovascular disease, type 2 diabetes and Alzheimer’s disease [39].

Overall, this study suggests the potential for SARM to contribute to IL-1β production and disease activity in RA. A limitation of this study was that it was not possible to directly modulate SARM expression in the patient monocytes as there are currently no inhibitors available and genetic manipulation of primary monocytes is not feasible due to their short viability in culture. However, the data demonstrate that SARM protein expression is lower in RA monocytes from patients with active disease and could promote RA pathogenesis by lowering the inhibitory action of SARM on TLR1/2-induced IL-1β production. Furthermore, these results reveal a possible mechanism by which anti-TNF-α therapies affect IL-1β levels in RA patients.

## Supplementary Material

keab162_Supplementary_Data
