# Supplementary material for: Expression of sterile-α and armadillo motif containing protein (SARM) in rheumatoid arthritis monocytes correlates with TLR2-induced IL-1β and disease activity
Source: Rheumatology (Oxford). 2021 Feb 19;60(12):5843–53. doi: 10.1093/rheumatology/keab162 (PMC8645275; doi:10.1093/rheumatology/keab162)
Supplement: keab162_Supplementary_Data [file keab162_supplementary_data.zip › keab162-suppl_data/rhe-20-2690-File007.docx]

|  | **IL-1β** | | | **DAS28** | | |
| --- | --- | --- | --- | --- | --- | --- |
| **Variables** | **B Coefficient** | **95% CI** | **p-value** | **B Coefficient** | **95% CI** | **p-value** |
| ***SARM1*** | -56.276 | -90.286 to  -22.266 | 0.003 | -321.080 | -545.616 to  -96.544 | 0.008 |
| ***PYCARD*** | -0.068 | -0.320 to 0.183 | 0.575 | 0.399 | -1.010 to 1.809 | 0.558 |
| ***CASP1*** | -0.070 | -0.363 to 0.223 | 0.620 | -1.802 | -3.375 to -0.228 | 0.027 |
| ***NLRP3*** | 0.085 | -0.452 to 0.622 | 0.743 | -0.955 | -3.219 to 1.310 | 0.386 |
| **(Constant)** | 2.842 | 2.145 to 3.540 | <0.001 | 9.249 | 5.642 to 12.856 | <0.001 |

**Supplementary Table S1: Multivariable linear regression of IL-1β or DAS28 with *SARM1*, *PYCARD*, *CASP1* and *NLRP3.*** The multivariable linear regression analysis was performed with the log10 transformed IL-1β data. Abbreviations: IL-1β, interleukin-1β; DAS28, disease activity score 28; CI, Confidence Interval.

**Supplementary Figure S1. TLR1/2 induced IL-1β secretion and *SARM1* expression are not influenced by glucocorticoid or methotrexate treatment in RA monocytes.** (A) IL-1β secretion measured following 24h of stimulation with 100ng/ml Pam3CSK4 (n=28) and (B) *SARM1* basal expression (n=31) was measured in RA monocytes and compared between RA patients with or without methotrexate or glucocorticoid treatment.

**Supplementary Figure S2. SARM expression does not correlate with LPS or R-848 induced IL-1β secretion** **in RA monocytes.** IL-1β secretion was measured following 24h of stimulation with (A) 10ng/ml LPS or (B) 2µg/ml R-848 and correlated with the basal expression of *SARM1* (n=17) in monocytes from RA patients.
