# Supplementary figures and images for: Expression of sterile-α and armadillo motif containing protein (SARM) in rheumatoid arthritis monocytes correlates with TLR2-induced IL-1β and disease activity
Source: Rheumatology (Oxford). 2021 Feb 19;60(12):5843–53. doi: 10.1093/rheumatology/keab162 (PMC8645275; doi:10.1093/rheumatology/keab162)

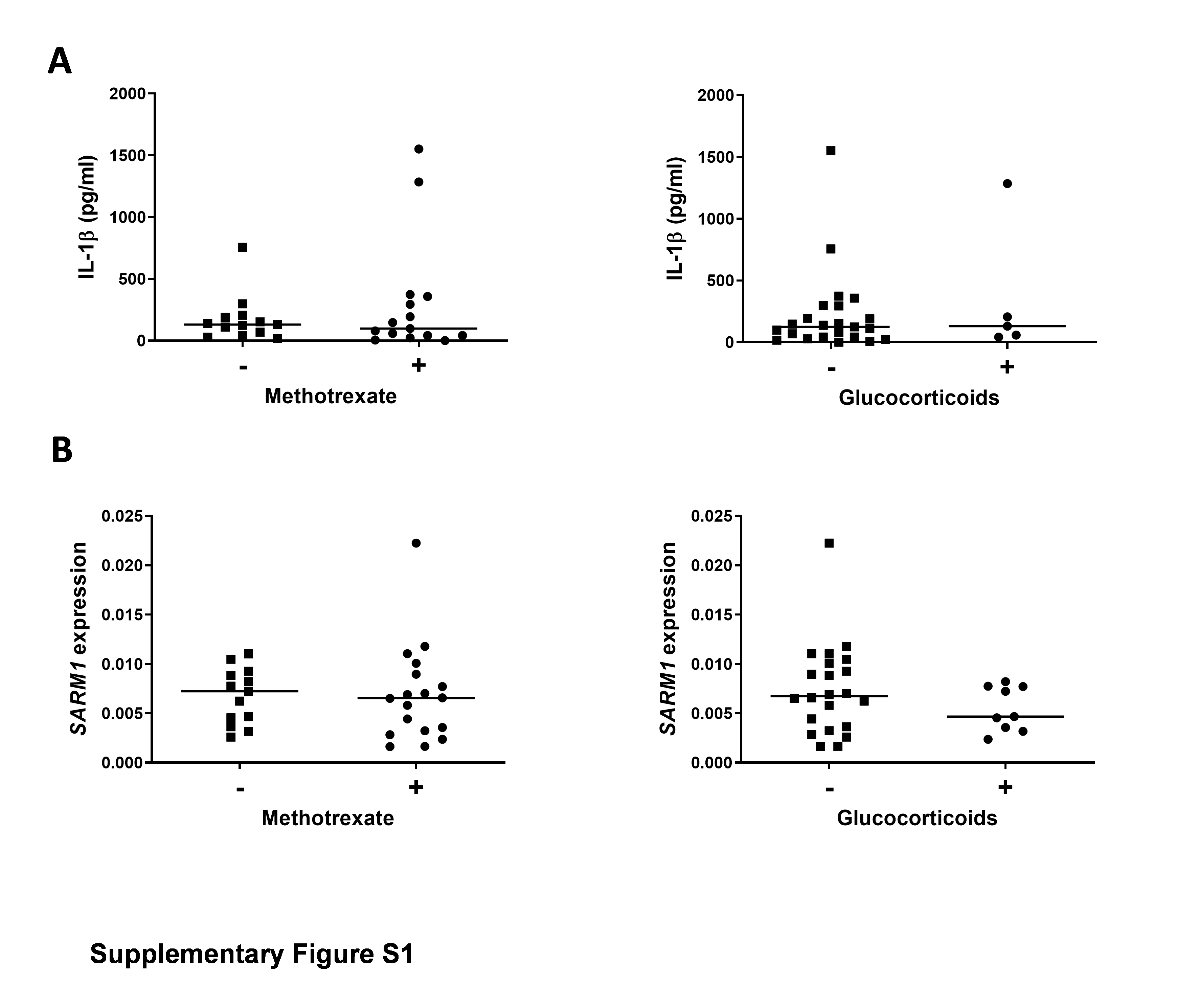

Supplement: keab162_Supplementary_Data [file keab162_supplementary_data.zip › keab162-suppl_data/rhe-20-2690-File008.tif]

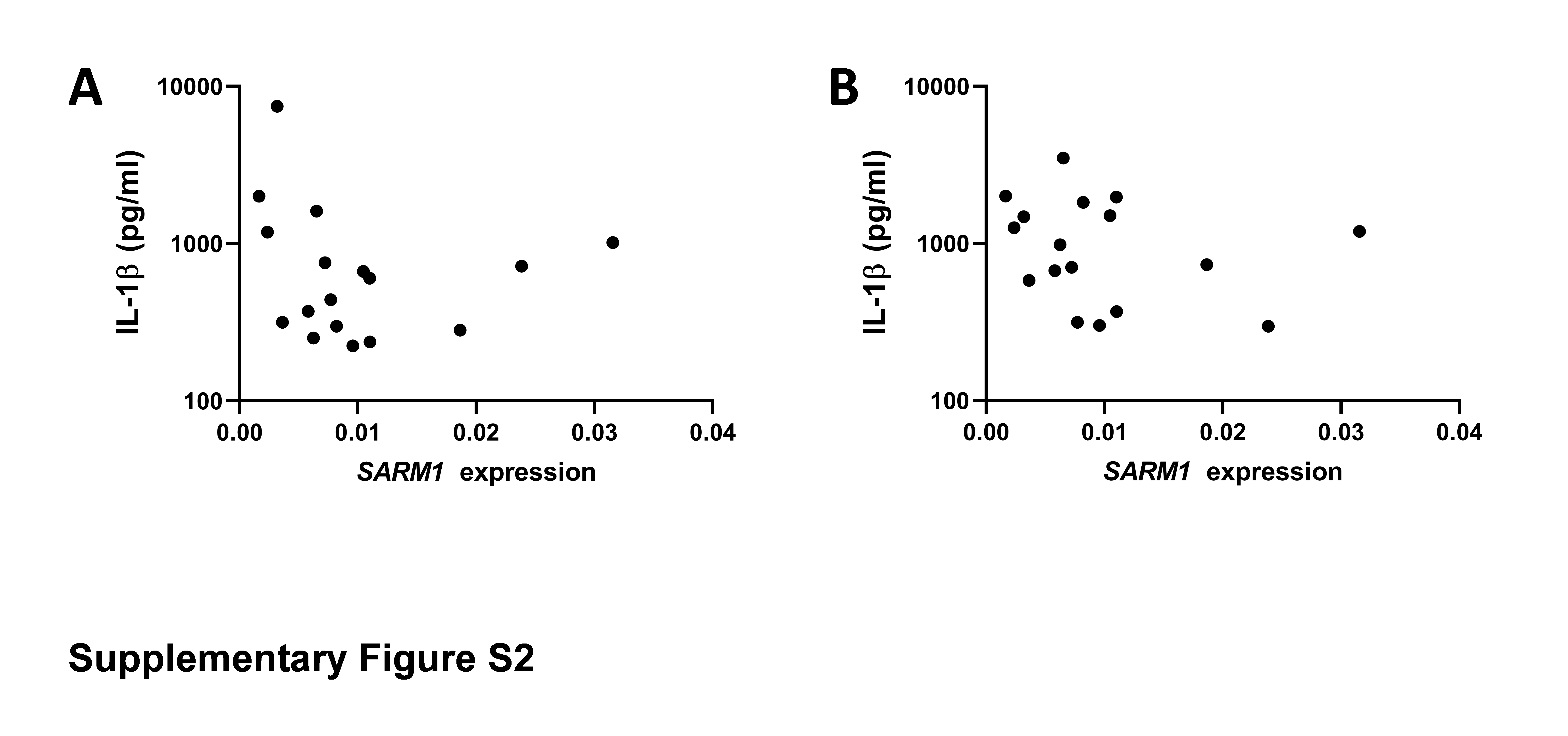

Supplement: keab162_Supplementary_Data [file keab162_supplementary_data.zip › keab162-suppl_data/rhe-20-2690-File009.tif]
